# An insulin-like signalling pathway model for *Fasciola gigantica*

**DOI:** 10.1186/s12917-024-04107-7

**Published:** 2024-06-08

**Authors:** Dongqi Wu, Yuqing Yang, Yankun Yang, Liang Li, Shishi Fu, Lei Wang, Li Tan, Xiuhong Lu, Weiyu Zhang, Wenda Di

**Affiliations:** 1 College of Animal Science and Technology, Guangxi University, Nanning, Guangxi China; 2 Wuhan Keqian Biology Limited Company, Wuhan, Hubei China; 3 Nanning Animal Disease Prevention and Control Center, Nanning, Guangxi China

**Keywords:** ESP, *Fasciola gigantica*, Insulin signalling pathway, Transcription, Trematode

## Abstract

**Background:**

The insulin/insulin-like signalling (IIS) pathway is common in mammals and invertebrates, and the IIS pathway is unknown in *Fasciola gigantica.* In the present study, the IIS pathway was reconstructed in *F. gigantica*. We defined the components involved in the IIS pathway and investigated the transcription profiles of these genes for all developmental stages of *F. gigantica*. In addition, the presence of these components in excretory and secretory products (ESPs) was predicted via signal peptide annotation.

**Results:**

The core components of the IIS pathway were detected in *F. gigantica*. Among these proteins, one ligand (*Fg*ILP) and one insulin-like molecule binding protein (*Fg*IGFBP) were analysed. Interestingly, three receptors (*Fg*IR-1/*Fg*IR-2/*Fg*IR-3) were detected, and a novel receptor, *Fg*IR-3, was screened, suggesting novel functions. *Fg14-3-3ζ*, *Fgirs*, and *Fgpp2a* exhibited increased transcription in 42-day-old juveniles and 70-day-old juveniles, while *Fgilp*, *Fgigfb*, *Fgsgk-1*, *Fgakt-1*, *Fgir-3*, *Fgpten*, and *Fgaap-1* exhibited increased transcription in metacercariae. *Fg*ILP, *Fg*IGFBP, *Fg*IR-2, *Fg*IR-3, and two transcription factors (*Fg*HSF-1 and *Fg*SKN-1) were predicted to be present in *Fg*ESPs, indicating their exogenous roles.

**Conclusions:**

This study helps to elucidate the signal transduction pathway of IIS in *F. gigantica*, which will aid in understanding the interaction between flukes and hosts, as well as in understanding fluke developmental regulation, and will also lay a foundation for further characterisation of the IIS pathways of trematodes.

**Supplementary Information:**

The online version contains supplementary material available at 10.1186/s12917-024-04107-7.

## Background

The insulin/insulin-like signalling (IIS) pathway is initiated by insulin and insulin-like growth factors and amplified by signalling cascades that are regulated by transcription factors, thereby regulating a range of biological processes [1]. When IIS is activated, downstream transcription factors are highly important for the communication and coordination of cells, tissues and organs [2, 3]. In mammals, IIS is involved in the regulation of lifespan, reproductive development, and metabolism [4, 5]. Research in *Caenorhabditis elegans* has revealed that the IIS pathway regulates lifespan, reproduction, and metabolism, suggesting that this pathway has a conserved function [6, 7]. In addition, IIS signalling plays a role in dauer development in *C. elegans* [8]. As insulin receptors are activated, transduced signals induce the phosphorylation of the transcription factor DAF-16 and subsequently promote the development of the worm under favourable circumstances [9, 10]. When unphosphorylated DAF-16 promotes downstream gene transcription, *C. elegans* can enter the dauer phase under unfavourable circumstances. In a study of *Schistosoma mansoni*, the upstream components of the IIS pathway were shown to potentially regulate fluke development and interaction with the host [11].

Research on the IIS pathway in *Fasciola gigantica* is lacking, and the IIS pathway model in *F. gigantica* is unknown. In-depth exploration of this pathway is helpful for exploring fluke development and fluke–host interactions, which may guide the prevention of fascioliasis. In addition, given the evolutionary conservation of the IIS pathway, studies regarding its role in *F. gigantica* may help to elucidate its function and regulation in other organisms and promote the treatment of metabolic diseases, such as diabetes and tumour [12, 13]. Fortunately, transcriptomic and genomic studies of *F. gigantica* have laid a solid foundation for further exploration. In this study, we explored the IIS pathway, mainly by (i) identifying insulin signal transduction components, (ii) examining their transcriptional profiles at different stages in the *F. gigantica* lifespan, and (iii) predicting their presence in excretory-secretory products (ESPs).

## Methods

### Identification of insulin signalling pathway components

Since the IIS pathway in *C. elegans* has been fully annotated (https://parasite.wormbase.org/index.html) and insulin pathway components have been partially identified in *Schistosoma japonicum*, the homologous components of the *F. gigantica* insulin signalling pathway were screened based on the corresponding components. BLAST searches were performed using known insulin pathway components to screen homologues of *F. gigantica* in nonredundant protein sequences, as well as the *F. gigantica* genome (PRJNA339660) and transcriptome (NCBI BioProject accession no. PRJNA230515) databases. Then, all screened components were mapped using BLAST (E-value cut-off: 10^− 5^) to verify that their regions were homologous to known insulin signalling components. All of the screened proteins were characterised by their primary amino acid sequence, and their structural and/or functional domains were inferred using bioinformatics tools (i.e., SignaIP5.0, TMHMM2.0, SWIMMMODEL, Expaxy, InterproScan and SMART) [14–16].

In addition, phylogenetic analyses were performed to analyse 3 receptors in the insulin signalling pathway of *F. gigantica*. Based on sequences from nematodes (*C. elegans* DAF-2), invertebrates (*Drosophila melanogaster* IR-1/IR-2), platyhelminths (*S. mansoni* IR-1/IR-2, *S. japonicum* IR-1/IR-2, *Fasciola hepatica* IR-1/IR-2/IR-3, *Fasciolopsis buski* IR-1/IR-2/IR-3, *Echinococcus multilocularis* IR-1/IR-2), and chordates (*Homo sapiens* IR-1/IR-2, *Mus musculus* IR-1/IR-2), the evolutionary relationships of *Fg*IR-1/*Fg*IR-2/*Fg*IR-3 were explored. Phylogenetic analyses of the aligned sequence data were conducted using the neighbour‒joining (NJ), maximum parsimony (MP), and maximum likelihood (ML) methods and the Jones–Taylor–Thornton (JTT) model in MEGA 11.0. The confidence intervals were evaluated using a bootstrap procedure with 1000 pseudoreplicates. A 50% cut-off value was used for the consensus tree [17].

### Analysis of differential transcription

Transcriptomic data from different developmental stages of *F. gigantica*, including the egg, miracidiae, rediae, cercariae, and metacercariae stages, as well as 42-day old juveniles, 70-day old juveniles, and adults, were previously generated by RNA-seq, using 3 samples from each stage and age group [18] and made publicly available (NCBI BioProject accession no.PRJNA350370). The sequences were aligned to the available *F. gigantica* genome sequence (accession number GWHAZTT00000000). The transcription of the genes were calculated by the fragments per kilobase per million reads (FPKM) method [19], which was used to compare the differences in gene transcription levels across different developmental stages. The false discovery rate (FDR) is a statistical method that was used to correct for *p* values. Genes with an adjusted *p* value < 0.05 according to DESeq were considered differentially transcribed genes [20]. The transcription data were analysed, clustered, and visualised using an online program (https://cloud.oebiotech.com/task/detail/heatmap/).

### Prediction of insulin signalling pathway components in ESPs

While classic secretory proteins were predicted by the SignalP 5.0, TargetP and TMHMM programs [21–23], noncanonical secretory proteins were predicted by the SecretomeP-2.0 program [24].

## Results

### Identification and characterisation of insulin signalling pathway components

The upstream components of the IIS pathway in *F. gigantica* were screened based on the *S. japonicum* insulin-like peptide and receptor components. One insulin-like peptide homologue (*Fg*ILP), one insulin-like molecular binding protein (*Fg*IGFBP), and 3 insulin receptors (*Fg*IR-1, *Fg*IR-2 and *Fg*IR-3) were identified (Table 1).

Fourteen homologous components downstream of the pathway in *C. elegans* were screened in *F. gigantica*, including 11 signal transduction proteins (*Fg*IRS, *Fg*AAP, *Fg*PI3K, *Fg*PDK-1, *Fg*AKT-1, *Fg*AKT-2, *Fg*SGK-1, *Fg*PTEN, *Fg*PP2A, *Fg*14-3-3ζ, and *Fg*DDL-1) and 3 transcription factors (*Fg*FOXO, *Fg*SKN-1 and *Fg*HSF-1) (Table 2).

**Table 1:** Upstream components of the IIS pathway

Protein	Accession no.	Homologues	Accession no.	Pairwise sequence similarity (%)
*Fg*ILP	PP157594	*Sj*ILP-1	AAW26714	35.88
*Fg*IGFBP	TPP67755	-	-	-
*Fg*IR-1	TPP62725	*Sj*IR-1	KAH8877623	59.23
*Fg*IR-2	TPP64585	*Sj*IR-2	KAH8876019	47.23
*Fg*IR-3	TPP63095	-	-	-

In the present study, one insulin ligand, *Fg*ILP, which encodes 137 aa and has 35.88% similarity to *Sj*ILP, was identified. InterScan analysis revealed that *Fg*ILP contains IGF family domains, which include the insulin B chain and insulin A chain. Like other insulin-like peptides, *Fg*ILP has a conserved cysteine motif, namely, the ‘CCXXXXCXXXXXXXC’ sequence (Additional file 1, Fig. S1).

*Fg*IGFBP encodes 435 aa and has 27.70% similarity to *Hs*IGFBP. *Fg*IGFBP contains an insulin-like growth factor binding protein region located at aa 59 to 116, and the IB domain is located at the C-terminus (Additional file 1, Fig. S1).

Three receptors, *Fg*IR-1, *Fg*IR-2 and *Fg*IR-3, were analysed, and each of them contained protein kinase binding regions and ATP binding sites. *Fg*IR-1 shares 58.37% similarity with *Sm*IR-1, and *Fg*IR-2 shares 50.51% similarity with *Sm*IR-2. *Fg*IR-3 shares 41.49% similarity with *Fg*IR-1 and shows 48.03% similarity with *Fg*IR-2. However, a BLAST search of *Fg*IR-3 revealed no homologues in trematodes (Additional file 1, Fig. S1).

**Table 2:** Downstream components of the IIS pathway

Protein	Accession no.	Homologues	Accession no.	Pairwise sequence similarity (%)
*Fg*IRS	TPP64173	*Ce*IST-1	AAC40865	30.17
*Fg*AAP	TPP59914	*Ce*AAP-1	AAF28335	30.93
*Fg*PI3K	TPP65574	*Ce*AGE-1	AAC47459	30.32
*Fg*PDK-1	TPP60389	*Ce*PDK-1a *Ce*PDK-1b	AAD42307 AAD42308	31.37
*Fg*AKT-1	TPP63762	*Ce*AKT-1	NP_001023647	48.56
*Fg*AKT-2	TPP59085	*Ce*AKT-2	NP_001024612	60.98
*Fg*SGK-1	TPP63805	*Ce*SGK-1	NP_001367134	57.54
*Fg*PTEN	TPP57329	*Ce*DAF-18	AAD03420.1	37.37
*Fg*PP2A	TPP62937	*Ce*PPTR-1	NP_507133	60.57
*Fg*14-3-3ζ	TPP62697	*Ce*PAR-5 *Ce*FTT-2	CAA98138.1 NP_509939	62.66 64.68
*Fg*DDL-1	TPP66199	*Ce*DDL-1 *Ce*DDL-2	CCD64976 CAB07688	40.32 -
*Fg*FOXO	TPP61970	*Ce*DAF-16	AAC47803	35.48
*Fg*HSF-1	TPP66402	*Ce*HSF-1	CAA22146	30.33
*FgS*KN-1	TPP67060	*Ce*SKN-1	CDK13323	39.13

Eleven intracytoplasmic components, including *Fg*IRS, *Fg*AAP, *Fg*PI3K, *Fg*PDK-1, *Fg*AKT-1, *Fg*AKT-2, *Fg*SGK-1, *Fg*PTEN, *Fg*PP2A, *Fg*14-3-3ζ and *Fg*DDL were investigated. *Fg*AAP encodes 1171 aa, is 30.93% similar to *Ce*AAP-1, and contains a protein kinase C domain and src homology 2 domain (SH2 domain). *Fg*PI3K encodes aa 1304, which includes a p85-binding domain and a Ras-binding domain. In addition, the catalytic domain of class I phosphoinositide 3-kinase is located at the C-terminus. *Fg*PDK-1 shares 31.71% similarity with *Ce*PDK-1 and contains a serine/threonine kinase catalytic site and a PH domain (Additional file 1, Fig. S2).

As protein kinases, *Fg*AKT-1 and *Fg*AKT-2 encode 957 and 278 aa, respectively, each of which contains a protein kinase domain. *Fg*SGK-1, which encodes a sequence of 350 aa, contains a protein kinase domain and a S–TK–X domain. *Fg*PTEN encodes 646 aa and contains the C2 domain of the PTEN tumour-suppressor protein (PTPc domain). *Fg*PP2A shares 60.57% similarity with *Ce*PPTR-1, which consists of a protein phosphatase 2 A regulatory B subunit. As a homologue of *Ce*FTT-2 and *Ce*PAR-5, *Fg*14-3-3ζ, which encodes 252 aa, shares 62.66% similarity with *Ce*PAR-5 and 64.98% similarity with *Ce*FTT-2, and consists of 14-3-3 domains. *Fg*DDL-1, which encodes 91 aa, shares 40.32% similarity with *Ce*DDL-2 and contains a WASH complex subunit homologue domain. Three transcription factors, *Fg*FOXO, *Fg*SKN, and *Fg*HSF were also identified. *Fg*FOXO encodes 987 aa and consists of conserved FH-FOXO motifs. *Fg*SKN-1 shares 39.13% similarity with *Ce*SKN-1 and consists of the basic leucine zipper (bZIP) domain of bZIP transcription factors. *Fg*HSF-1, which encodes 258 aa and shows 30.33% similarity to *Ce*HSF-1, contains a heat shock transcription factor domain (Additional file 1, Fig. S2).

The genes were cloned using primers (Additional file 2, Table S1) as shown in the gel (Additional file 3, Fig. S1) and sequenced. The actual length of sequences were consistent with the transcriptome data.

### Phylogenetic analysis

**Fig. 1:**
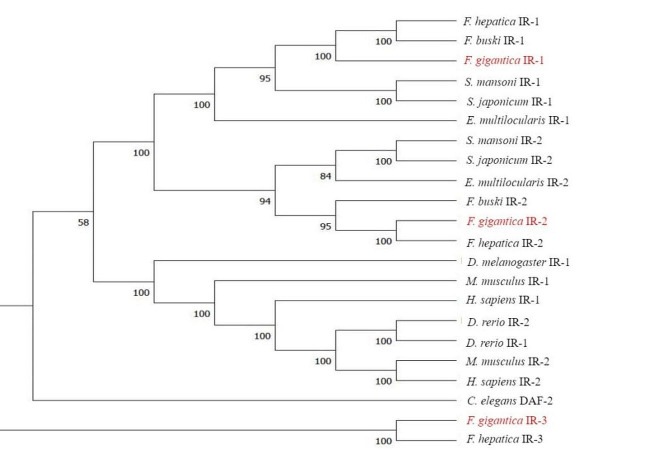
Phylogenetic relationships of insulin receptors in *F. gigantica* and homologues of 11 other species. A neighbour‒joining tree was constructed, and bootstrap values were shown below the branches. The sequences used and their GenBank accession numbers were as follows: *F. hepatica* (IR-1, THD26449; IR-2, THD19358; IR-3, THD24184); *F. gigantica* (IR-1, TPP62725; IR-2, TPP64585; IR-3, TPP63095. ); *F. buski* (IR-1, KAA0195064; IR-2, KAA0201248); *S. mansoni* (IR-1, AAN39120; IR-2, AAV65745); *S. japonicum* (IR-1, ACT20714; IR-2, ACT20715); *C. elegans* (DAF-2, AAC47715); *E. multilocularis* (IR-1, CAD30260; IR-2, CDS42114); *H. sapiens* (IR-1, XP-047288401; IR-2, NP-001073285); *Danio rerio* (IR-1, NP-00111670; IR-2, NP-001136144; *D. melanogaster* (IR-1, NP-00113809); *M. musculus* (IR-1, NP-035962; IR-2, NP-034698).

Phylogenetic trees were constructed to explore the relationships of the 3 receptors (Fig. 1). The topologies of the NJ, MP and ML trees were concordant (Additional file 1, Fig. S3). *Fg*IR-1 was grouped with *Sm*IR-1 with 91% bootstrap support. *Fg*IR-2 was grouped with *Sm*IR-2 and *Em*IR-2, while *Fg*IR-3 and *Fh*IR-3 constituted an independent cluster.

### The IIS pathway in *F. gigantica*

**Fig. 2:**
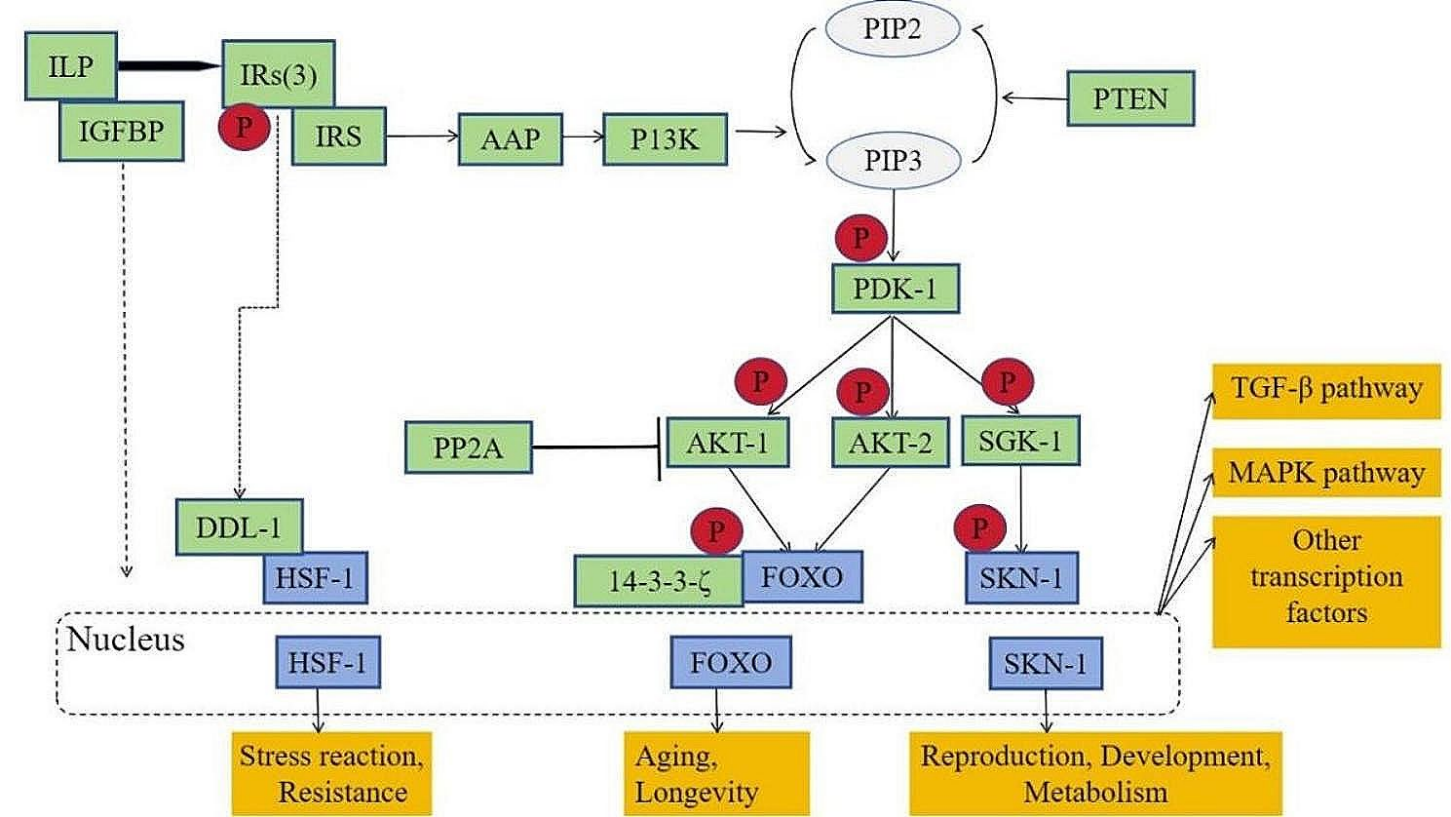
Schematic of the *F. gigantica* IIS pathway. After binding to the receptor, *Fg*ILP recruit the insulin receptor substrate IRS, leading to PI3K phosphorylation and promoting the conversion of PIP3 to PIP2. PIP3 signalling activates downstream kinase cascades and promotes *Fg*FOXO and *Fg*SKN-1 transcription factor translocation (solid line). The transcription factor *Fg*HSF-1 is activated by *Fg*DDL-1 and undergoes nuclear translocation (the dashed line indicates this). The core components of the insulin pathway are represented by green boxes; phosphorylation is represented by red circles, and the blue boxes represents the transcription factors. The insulin pathway regulates the transcription of various genes and subsequently regulates key processes, including stress reactions, resistance, longevity, ageing, reproduction, development, metabolism and other pathways, such as the TGF-β signalling pathway

Using the available information, we constructed an insulin-like signalling pathway for *F. gigantica* (Fig. 2). *Fg*ILP can bind to and activate the receptor *Fg*IR1/*Fg*IR2/*Fg*IR3, while *Fg*IGFBP can aggregate *Fg*ILP to reduce its free concentration, thereby regulating insulin signalling. *Fg*IR activation results in the recruitment of *Fg*PI3K. Then, the downstream kinases *Fg*PDK-1, *Fg*AKT-1, and *Fg*AKT-2 are activated, resulting in the phosphorylation of the transcription factor *Fg*FOXO. *Fg*14-3-3-ζ regulates its interaction with *Fg*FOXO to regulate *Fg*FOXO localisation. *Fg*SKN-1 is phosphorylated by *Fg*SGK-1, inhibiting its subcellular translocation to the nucleus. In addition to participating in a series of phosphorylation cascades, *Fg*TLP can also bind to and activate the receptor and subsequently phosphorylate *Fg*DDL-1 directly, in turn regulating the transcription of *Fg*HSF-1.

In the process of signal transduction, *Fg*PTEN acts as an activator to promote the transformation of PIP3 to PIP2, inducing the phosphorylation of *Fg*PDK-1 and promoting the posttranscriptional translation of transcription factors. As an antagonist, *Fg*PP2A can bind to *Fg*AKT, inhibiting its phosphorylation and subsequent signal transduction. Numerous downstream target genes are involved in various processes, such as stress reactions, longevity, reproduction and the activation of other pathways.

### Transcript abundance based on RNA-seq analysis

**Fig. 3:**
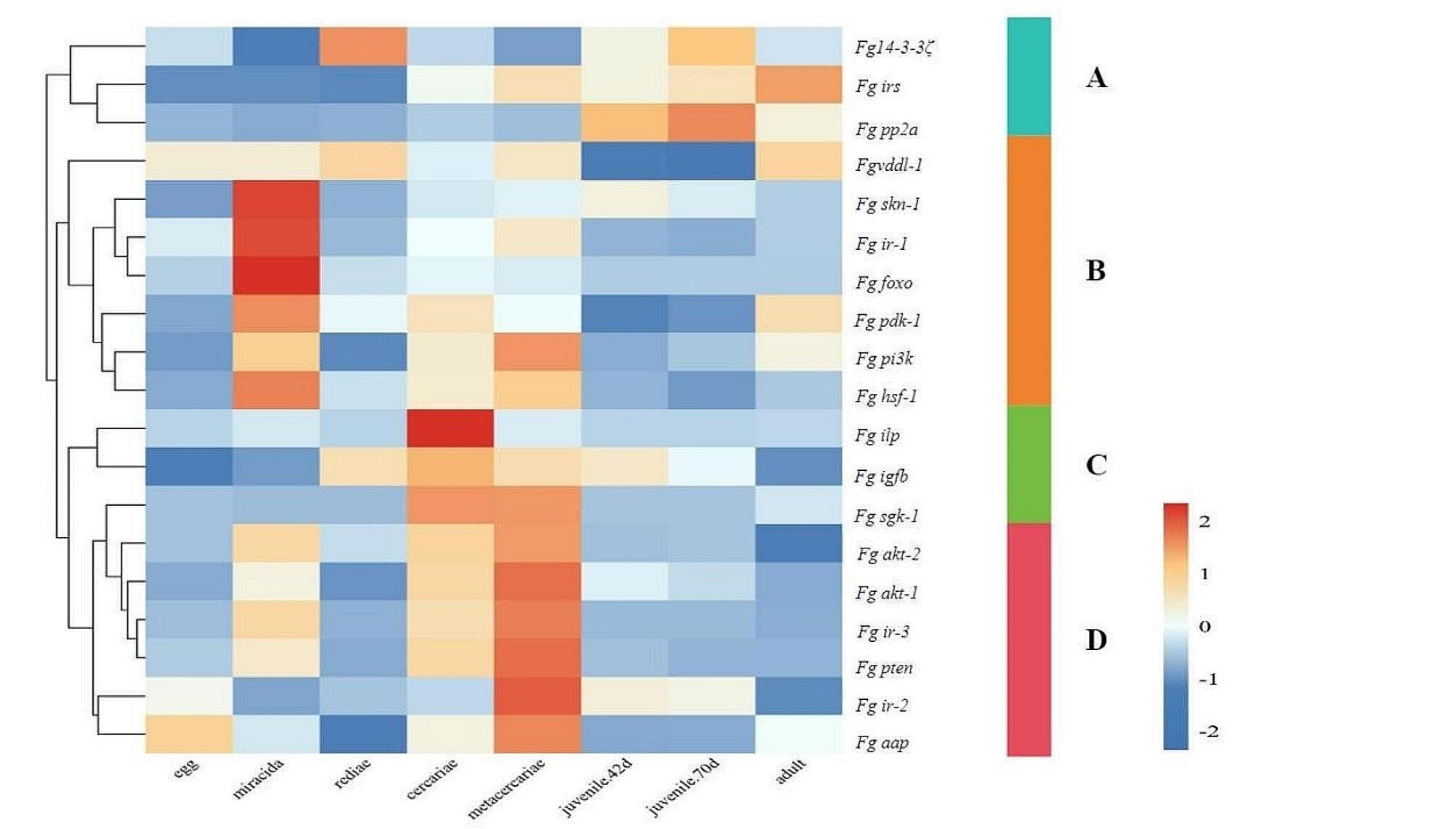
Transcription profile of insulin components during the lifecycle of *F. gigantica*. A graphical representation of the 19 components based on the FPKM values is displayed as a heatmap (upregulation is represented in red, and downregulation is represented in blue)

Among the 19 genes, group A genes (5 genes, including *Fg14-3-3-ζ*, *Fgir-2*, *Fgirs*, *Fgpp2a*, and *Fgddl-1*) were highly transcribed in 42-day-old juveniles and 70-day-old juveniles (Fig. 3). Group B (6 genes, including *Fgskn-1*, *Fg*p*dk-1*, *Fgir-1*, *F*g*foxo*, *F*g*hfs-1*, and *Fgpi3k*) exhibited increased transcription in miracidiae, while group C (3 genes, including *Fgaap*, *Fgsgk-1*, and *Fgilp*) exhibited increased transcription in cercariae and metacercariae. Group D (4 genes, including *Fgakt-1*, *Fgakt-2*, *Fgir-3*, and *Fgpten*) exhibited increased transcription in metacercariae.

Apart from *F*g*ir-2* and *Fgpdk-1*, 17 genes involved in the insulin signalling pathway were significantly differentially transcribed across the eight life stages of *F. gigantica* (Additional file 4, Table S2).

### The presence of *F. gigantica* insulin signalling pathway components in ESPs

Among the 19 components of the IIS, 8 were predicted to be present in ESPs. While *Fg*ILP, *Fg*IGFBP, and *Fg*PTEN were predicted to be classic secretory proteins, *Fg*IR-2, *Fg*IR-3, *Fg*DDL-1, *Fg*HSF-1, and *Fg*SKN-1 were predicted to be noncanonical secretory proteins.

## Discussion

In this study, we constructed the IIS pathway, analysed the transcription profiles of the components, and predicted their presence in ESPs. Since the components of the IIS pathway have been poorly studied in trematodes, the functions of *F. gigantica-*derived components were speculated, and further studies are needed to verify their specific roles.

Multicellular communication is a key factor in the survival of parasitic helminths. The hormone host–worm cross-communication hypothesis suggests that the endocrine and paracrine hormone systems of mammals (even invertebrates) can affect the physiology, development, and survival of metazoan parasites by stimulating the evolutionarily conserved signalling system [25]. According to previous studies, the insulin signalling pathway is widely involved in the reproductive development of parasitic helminths and host–parasite interactions [26]. As an endocrine-related pathway, the insulin signalling pathway of *F. gigantica* may have intrinsic and extrinsic functions, and exploring this pathway will deepen the understanding of fluke development, as well as the interaction between flukes and hosts, to guide the prevention of fascioliasis and the treatment of metabolic diseases.

In *S. japonicum*, one ligand, *Sj*ILP, was detected. Immunohistochemistry has shown that *Sj*ILPs are abundantly expressed in the basement membranes and ovaries of adult worms [27]. Du et al. confirmed that *Sj*ILP can bind to ADP, which is the main agonist of platelet recruitment, indicating that *Sj*ILP functions in glucose metabolism [28]. In *Taenia solium*, two ILP sequences were cloned, and *Ts*ILP was found to be localised to the ovary, possibly related to reproduction [29]. Here, in *F. gigantica*, one ligand homologue, *Fg*ILP, was screened, and *Fg*ILP may also be localised to reproductive organs, participating in the reproductive development and glucose metabolism of flukes. Mammalian IGFBP can bind to ILP and regulate its bioavailability. In addition, IGFBP has multiple proteolytic enzyme binding sites and can be broken down into multiple fragments during metabolism, suggesting that its role is independent of the ILP molecular network [30, 31]. Similarly, *Fg*IGFBP may also bind to *Fg*ILP and be involved in a role independent of the *Fg*ILP molecular network.

Two insulin receptors were characterised in both *S. japonicum* and *E. multilocularis*, and the two receptors differs in terms of structural motifs and tissue localisation. In terms of structure, *Sm*IR-1 and *Sm*IR-2 show 25.6% similarity, and the LBD and FN3 domains are greatly altered, indicating that they can be activated in different ways by binding to various ligands. *Sm*IR-1 is localised to the muscle, intestinal epithelia, and basement membrane teguments of adult females and is also associated with the glucose transporters SGTP1 and SGTP4, suggesting that it participates in glucose uptake [32]. The structural characteristics of *Em*IR-1 and *Em*IR-2 are similar to those of *Sm*IR-1 and *Sm*IR-2. RT‒PCR analysis revealed that *Em*IR-1 is transcribed in both protoscoleces and echinococcosis, mainly in glycogen storage cells, indicating that *Em*IR-1 may be related to glucose metabolism [33]. The phylogenetic tree showed that *Fg*IR-1 clustered on the same branch as *Sj*IR-1 and *Em*IR-1. Therefore, *Fg*IR-1 may be involved in glucose metabolism.

In addition to its intrinsic role, insulin-like signalling is involved in parasite–host communication. In *S. japonicum*, *Sj*IR-2 can specifically bind to host insulin; further studies have suggested that *S. japonicum* can use host insulin for its growth and development and regulate cell proliferation and differentiation through the same pathway as host cells [34]. You et al. reported that genes related to the *S. japonicum* insulin signalling pathway, such as *Sj*IR-2, were upregulated in the presence of human insulin [35]. After co-culturing schistosomes with host insulin and schistosomes for 24 h, microarray analysis showed that the transcription of two genes in the MAPK pathway was up-regulated, indicating that host insulin can act on the trematode insulin pathway and participate in the information exchange between trematodes and hosts in combination with the MAPK signaling pathway [36]. In mouse infection models of *E. multilocularis*, the addition of host insulin can promote the phosphorylation of *Em*IR-2 in vesicles, significantly altering the phosphorylation profile of PI3K/Akt signalling pathway components in vesicles and leading to the activation of downstream signalling pathways [37]. Although the *E. multilocularis* genome encodes ILP, yeast two-hybrid experiments revealed that *Em*IR-1 does not interact with *Em*ILP but rather that the parasite-derived receptor *Em*IR-1 strongly binds to host proinsulin [38]. As *F. gigantica* is a multicellular organism, its parasitism may be regulated by signalling networks. *Fg*IR-2 is predicted to be expressed in *Fg*ESP, and it may play a similar role as *Sj*IR-2 and *Em*IR-2 in parasite–host interactions; these proteins may activate and amplify the downstream signals of the parasite by binding to the insulin of the host and promoting reproductive development. *Fg*IR-3 was clustered on a separate branch independent of IR-1/IR-2 homologues, and its homologues were not found in other flukes, including *E. multiloculari*s, *S. japonicum*, and *S. mansoni*. *Fg*IR-3 may have evolved in *Fasciola* to adapt to parasitism, and further study of *Fg*IR-3 is necessary to determine its role in the development and parasitism of *F. gigantica*.

*Fg*IRS, *Fg*PI3K, *Fg*APP, *Fg*AKT-1, *Fg*AKT-2, *Fg*14-3-3-ζ, and *Fg*DDL-1 were also screened as cytoplasmic signal transduction components. In *S. mansoni*, an AKT isoform has been characterised, and blocking *Sm*AKT reduces male–female cohesion and decreases the oviposition rate [39]. *Fg*AKT-1 and *Fg*AKT-2 may also participate in the reproductive process.

Two regulatory molecules involved in the insulin signalling pathway were screened in *F. gigantica*. The homologues of *Ce*DAF-18 and *Fg*PTEN were analysed. In *C. elegans*, DAF-18 controls dauer development and lifespan by inhibiting the phosphorylation of PIP3, and DAF-18 also acts as a negative regulator of DAF-2 and AGE-1 [40, 41]. Similarly, *Fg*PTEN may act as an antagonist to promote the longevity of *F. gigantica*. Moreover, PPTR-1 acts as an inhibitor of the IIS pathway in *C. elegans*, and its homologues in humans and *Drosophila* can restrain the activation of AKT [42]. As a homologue of *Ce*PPTR-1, *Fg*PP2A may also inhibit insulin signalling transduction by blocking AKT phosphorylation.

In *F. gigantica*, three transcription factors, *Fg*FOXO, *Fg*SKN-1, and *Fg*HSF-1, were identified. Forkhead box O (FOXO) proteins constitute a subfamily of transcription factors that are conserved across invertebrates and mammals, and studies have consistently shown that FOXO proteins are important determinants of ageing and longevity. In *C. elegans*, FOXO is closely related to the stress response and cell cycle [43, 44]. In *Drosophila*, the upregulation of dFOXO in the bodies of adults is sufficient to promote longevity and antioxidative stress [45]. Here, *Fg*FOXO may also play roles in the ageing and longevity in *F. gigantica*. SKN-1 mediates the transcription of genes involved in detoxification and stress responses [46] and can also promote protein homeostasis by regulating proteasome production [47], which helps to extend lifespan [48]. *Fg*SKN-1 may also regulate the detoxification and stress response of *F. gigantica*. HSF-1 is a conserved regulator of heat-shock-induced gene transcription and regulates the overall stress response and stress resistance. In *C. elegans*, HSF-1 deficiency accelerates tissue ageing and shortens the lifespan [49]. In addition, DDL-1/2 can negatively regulate the activity of HSF-1 and regulate the stress response [50]. Therefore, it can be speculated that *Fg*SKN-1 may regulate the stress response and stress resistance of *F. gigantica*.

In *C. elegans*, the insulin signalling pathway is thought to play a role in dauer development. Further studies have demonstrated the similar function of this pathway in V clade parasitic nematodes; that is, the pathway promotes development from the iL3 stage, analogous to the dauer stage of *C. elegans*, to the parasitic stage [51]. In the present study, the B, C, and D group genes shown in Fig. 3 exhibited increased transcription in metacercariae. As metacercariae are a stage of flukes awaiting host development that are resistant to the external environment, which is analogous to the dauer stage of *C. elegans*, the insulin signalling pathway may promote the transition of metacercariae to the parasitic stage. However, this hypothesis needs further exploration.

## Conclusion

The insulin signalling pathway of *F. gigantica* was reconstructed, and the transcription profiles of these genes at different developmental stages were studied. In addition, the presence of these components in ESPs was explored, which provides a reference for future research on fluke development and *F. gigantica*–host interactions and promotes the exploration of other signalling pathways involved in this neglected tropical pathogen.

### Electronic supplementary material

Below is the link to the electronic supplementary material.

Supplementary Material 1

Supplementary Material 2

Supplementary Material 3

Supplementary Material 4

## Data Availability

All data analysed during this study are included in this published article. Zhang XX, Cong W, Elsheikha HM, Liu GH, Ma JG, Huang WY, Zhao Q, Zhu XQ. De novo transcriptome sequencing and analysis of the juvenile and adult stages of Fasciola gigantica. Infect Genet Evol. 2017.
