# Supplementary material for: An insulin-like signalling pathway model for Fasciola gigantica
Source: BMC Vet Res. 2024 Jun 8;20:252. doi: 10.1186/s12917-024-04107-7 (PMC11162077; doi:10.1186/s12917-024-04107-7)

Fig. S1. Domain annotation of upstream components of the insulin signalling pathway

*Fg*ILP


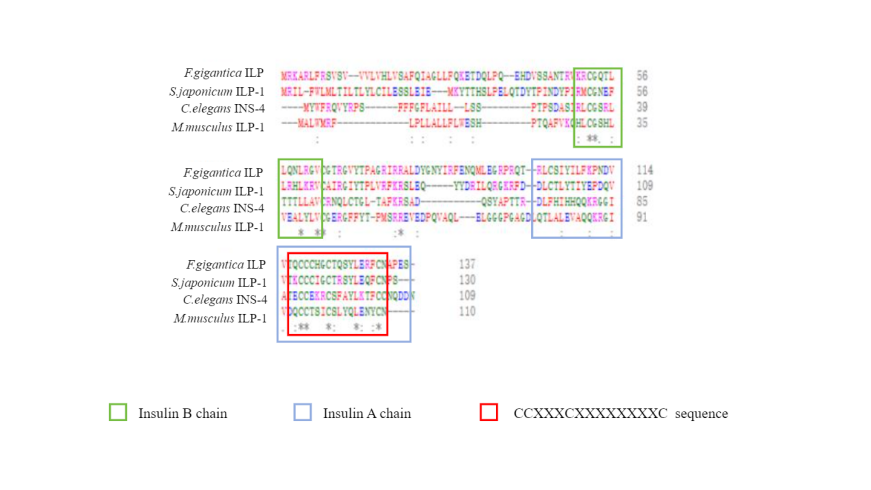


*Fg*IGFBP


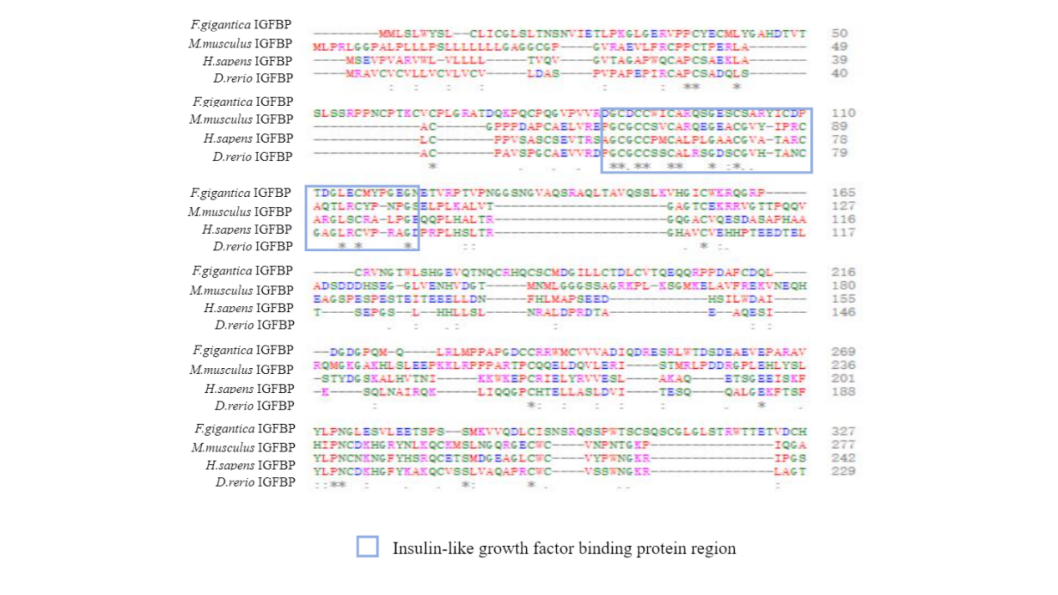


*Fg*IR


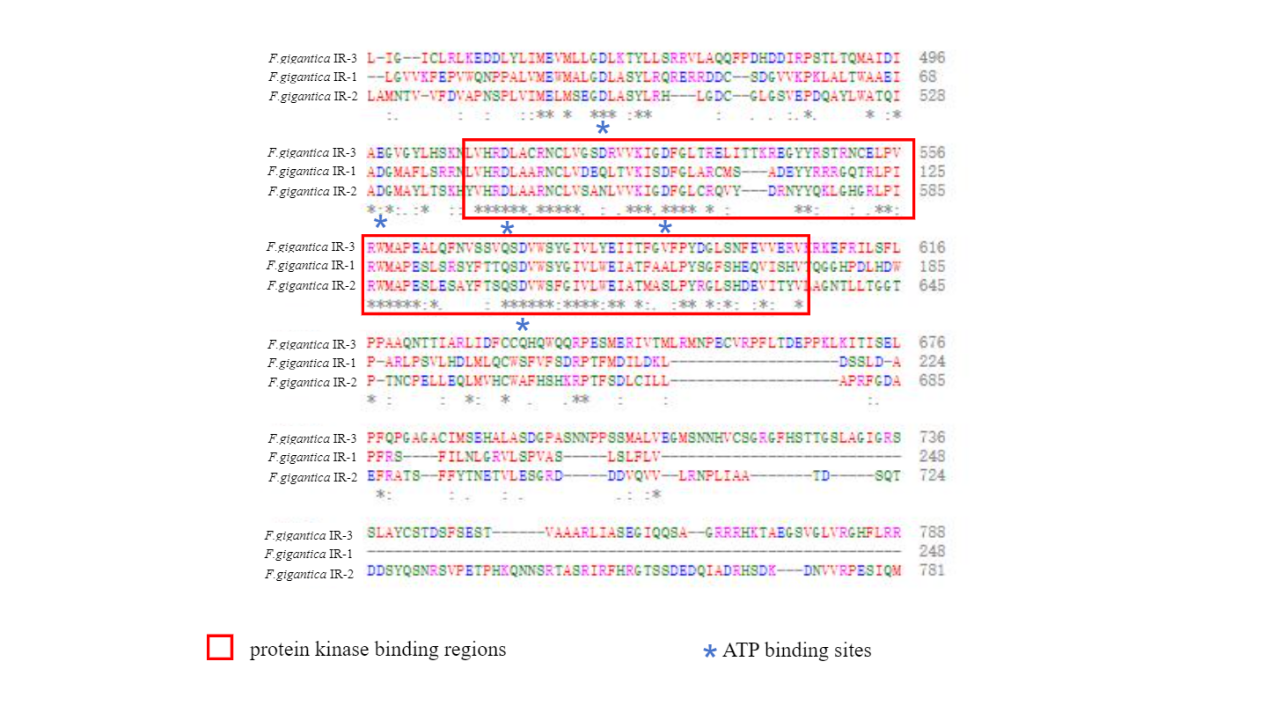


*Fg*IRS


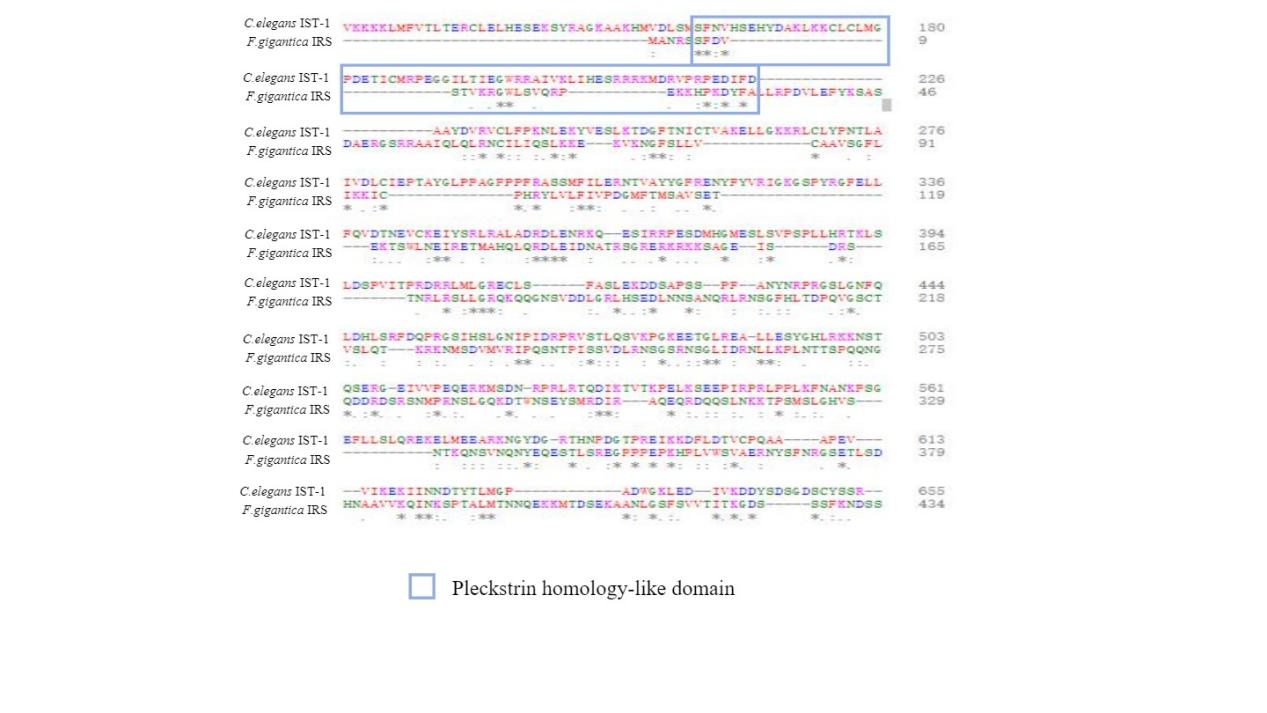


Fig. S2. Domain annotation of downstream components of the insulin signalling pathway

*Fg*AAP


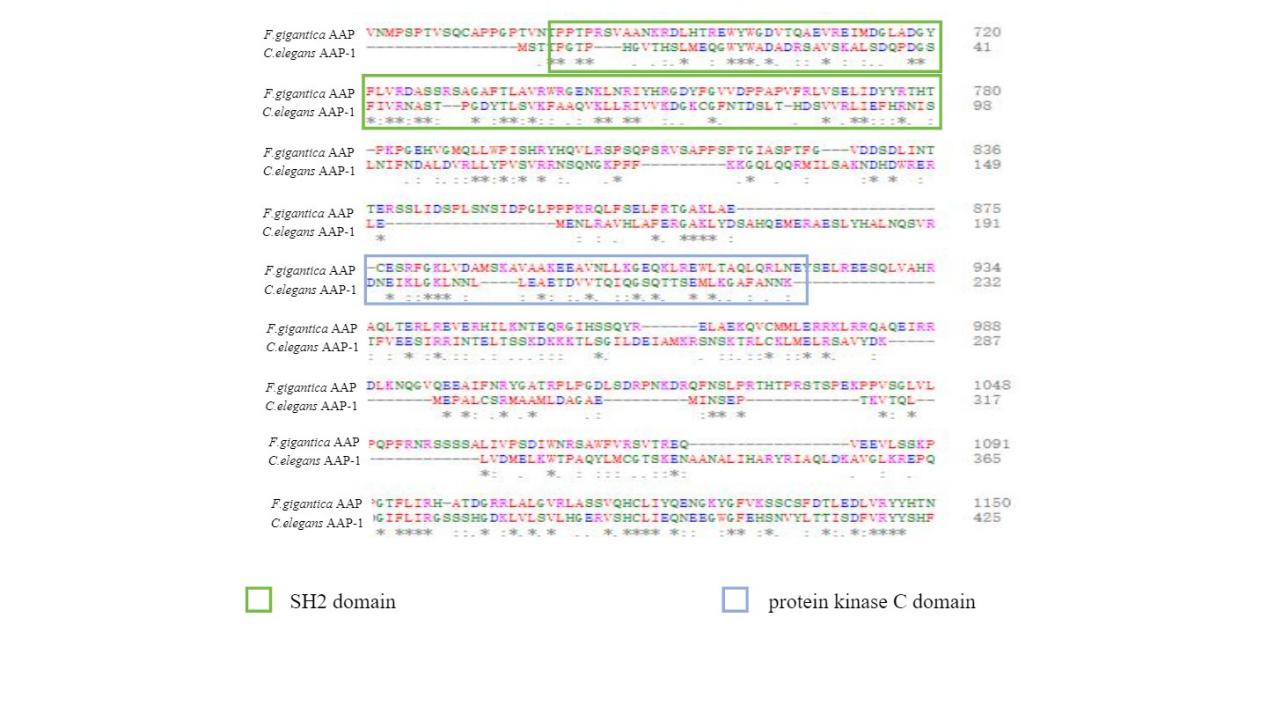


*Fg*PI3K


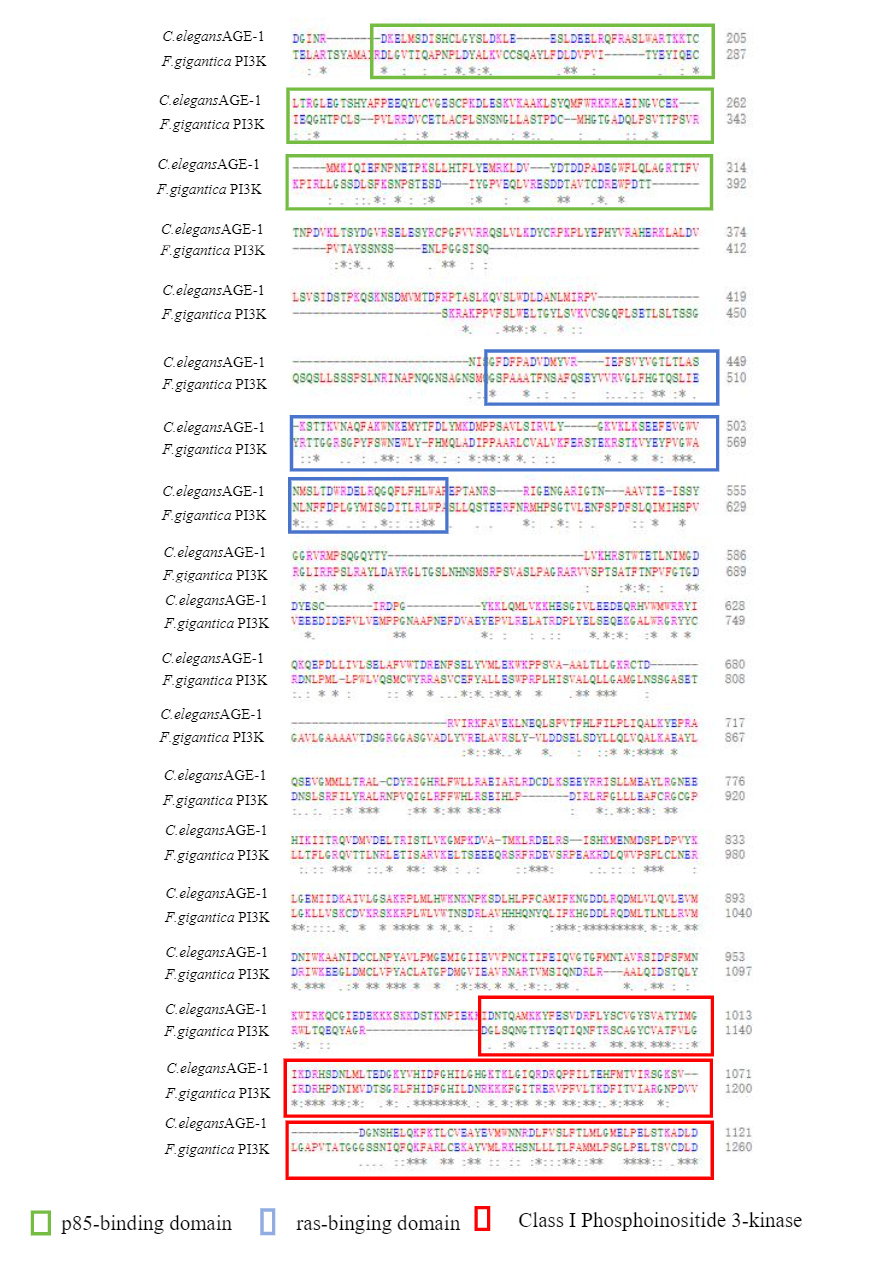


*
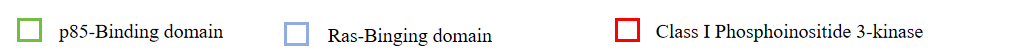
*

*Fg*PDK-1

*
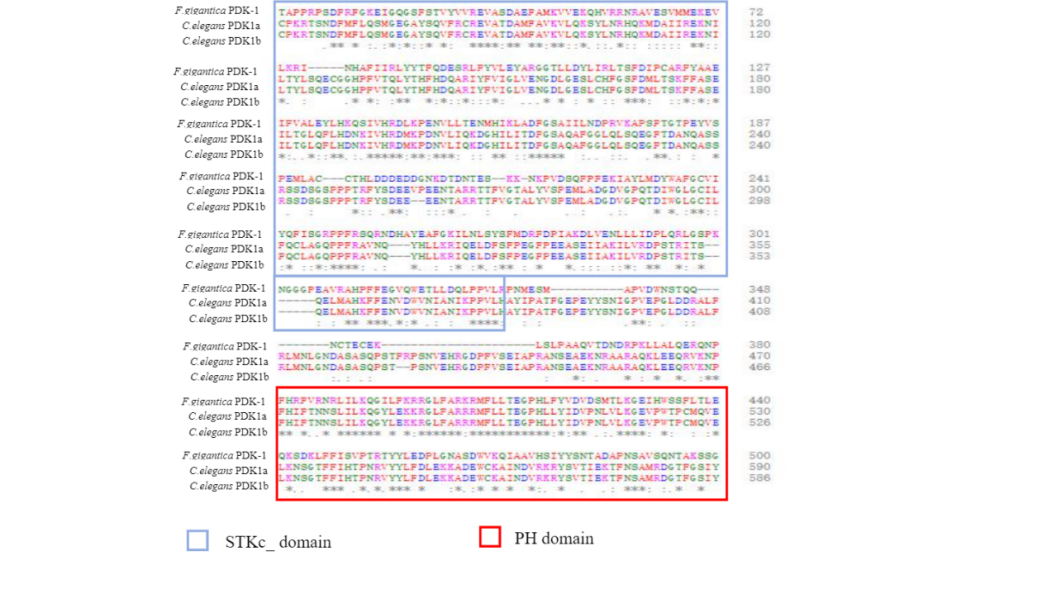
*

*Fg*AKT-1

*
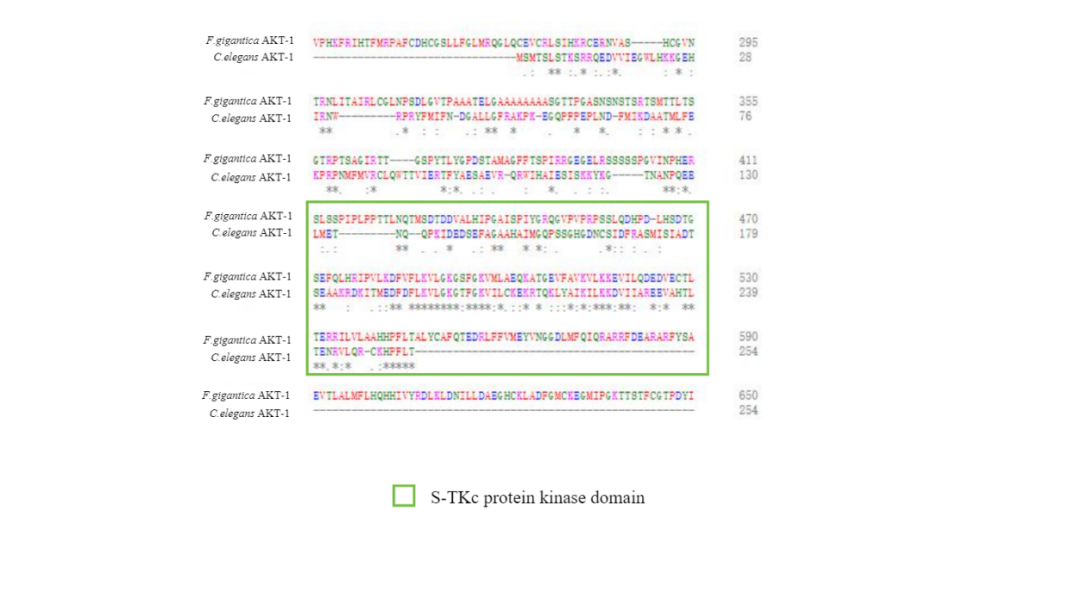
*

*Fg*AKT-2


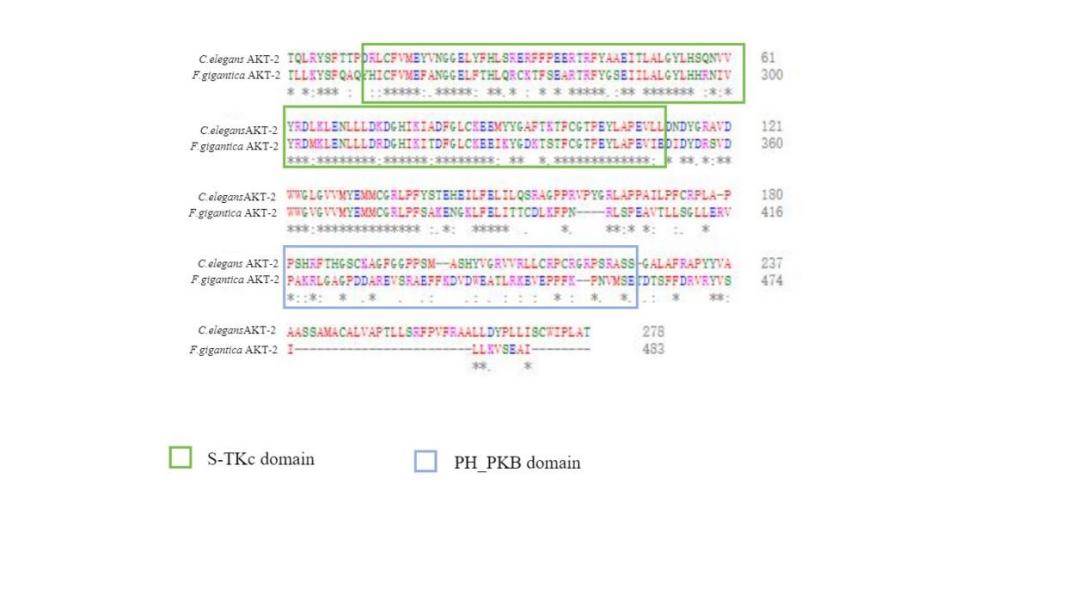


*Fg*SGK-1

*
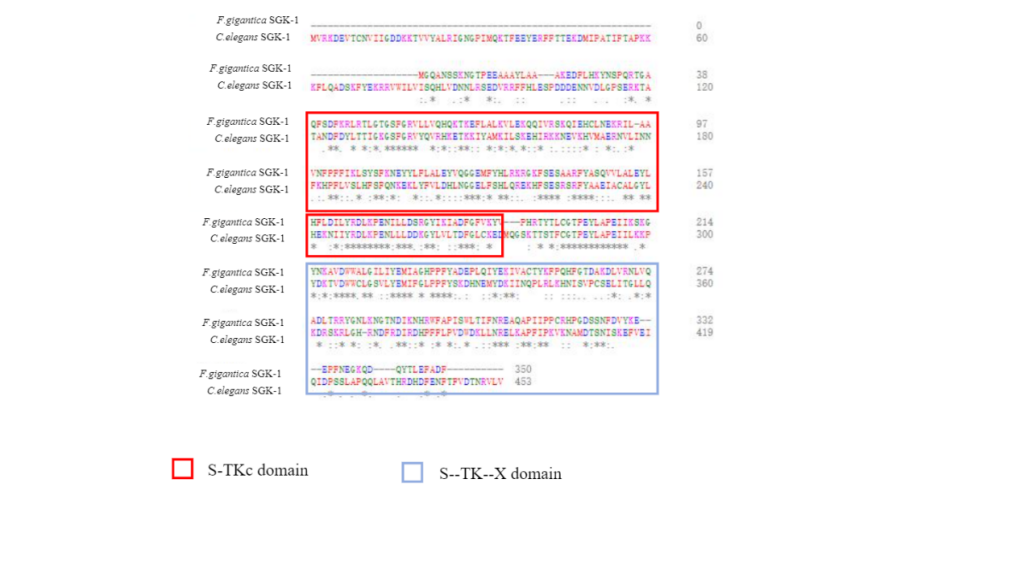
*

*Fg*PTEN

*
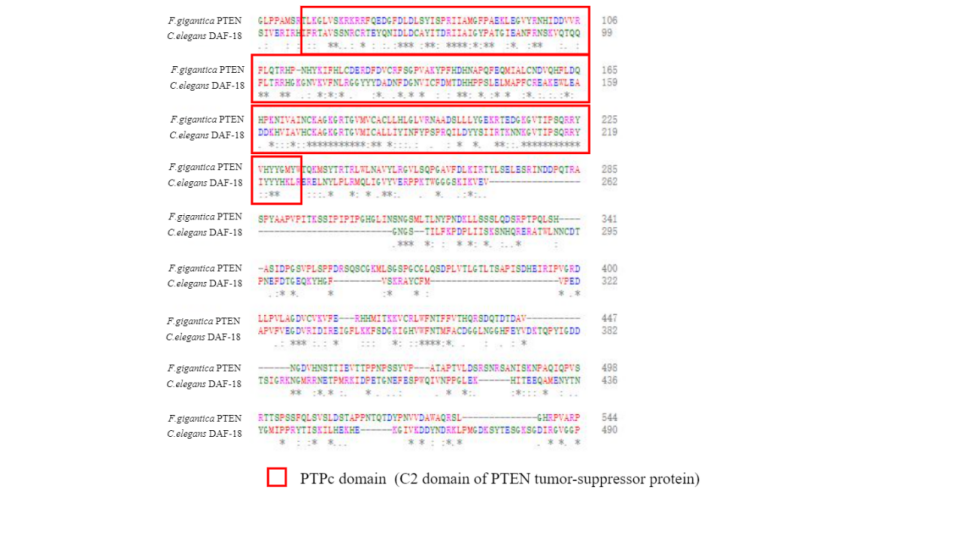
*

*Fg*PP2A


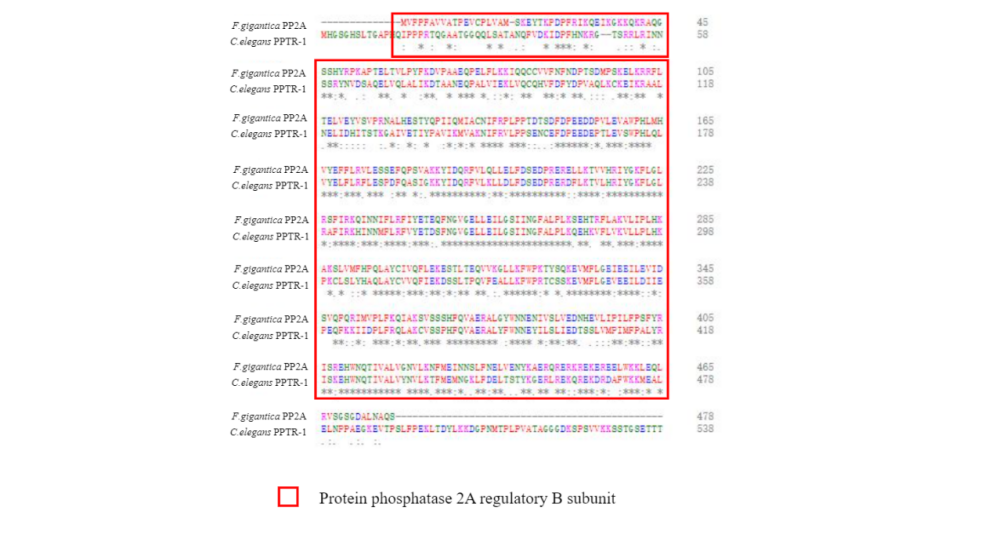


*Fg*14-3-3ζ

*
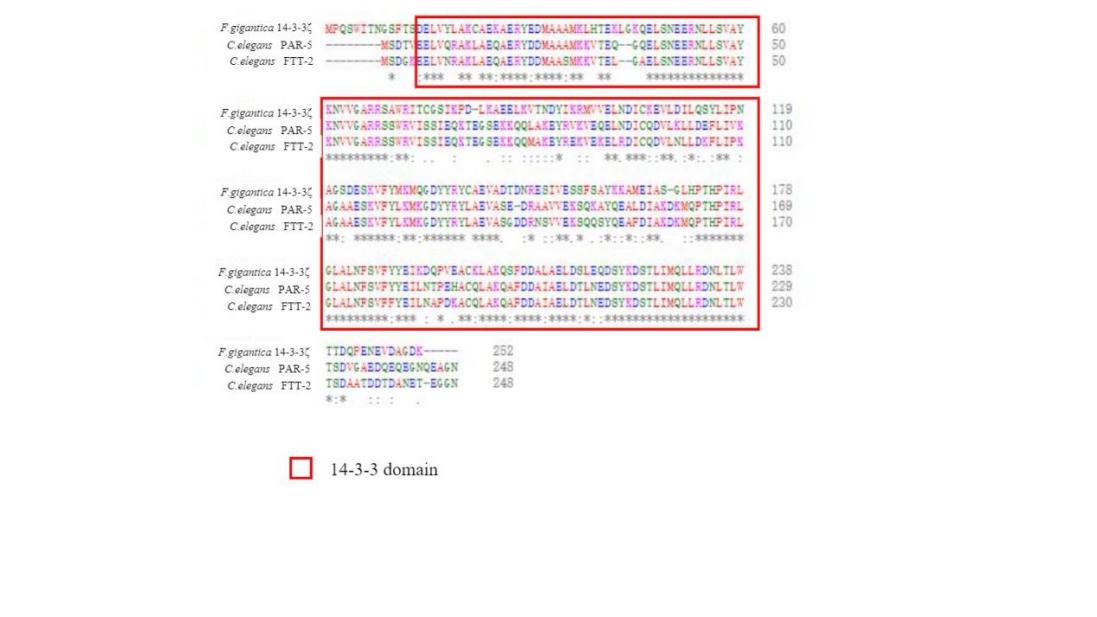
*

*Fg*DDL-1

*
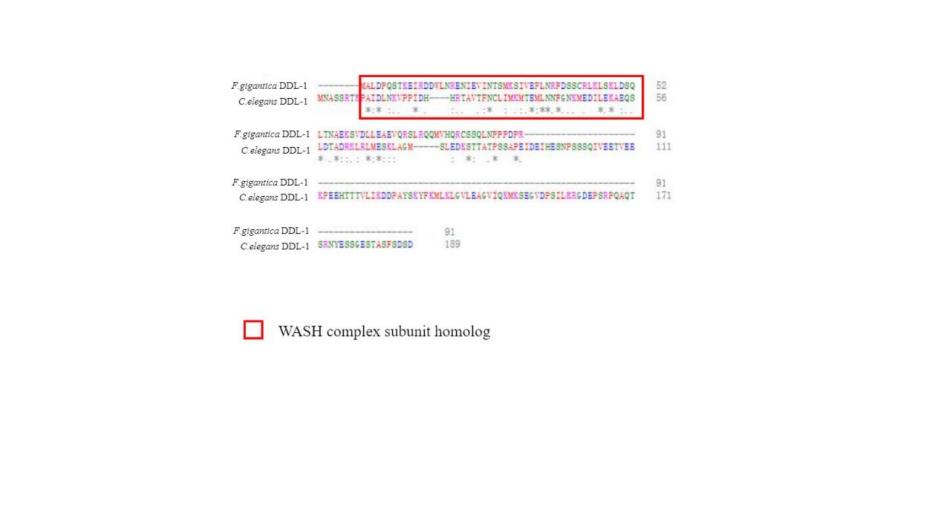
*

*Fg*FOXO

*
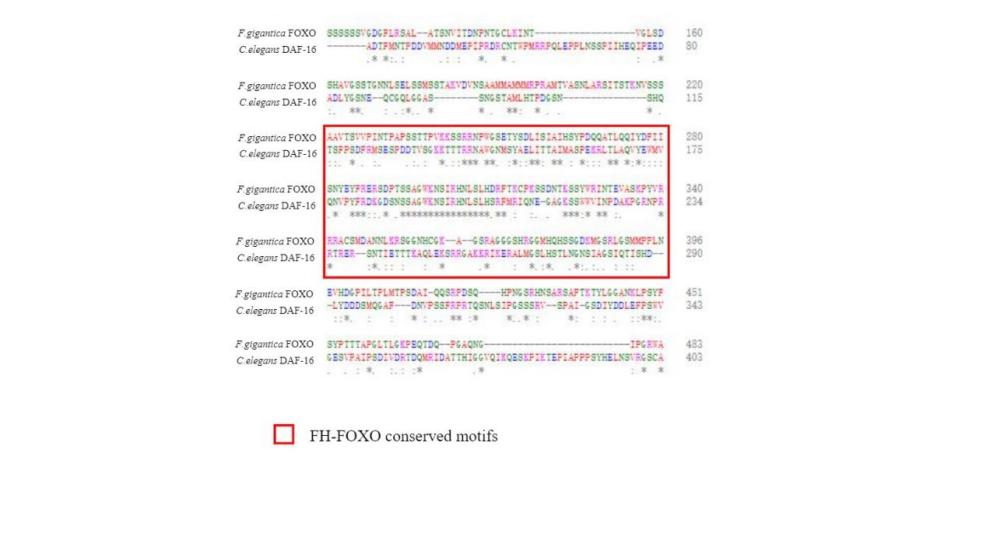
*

*Fg*SKN-1

*
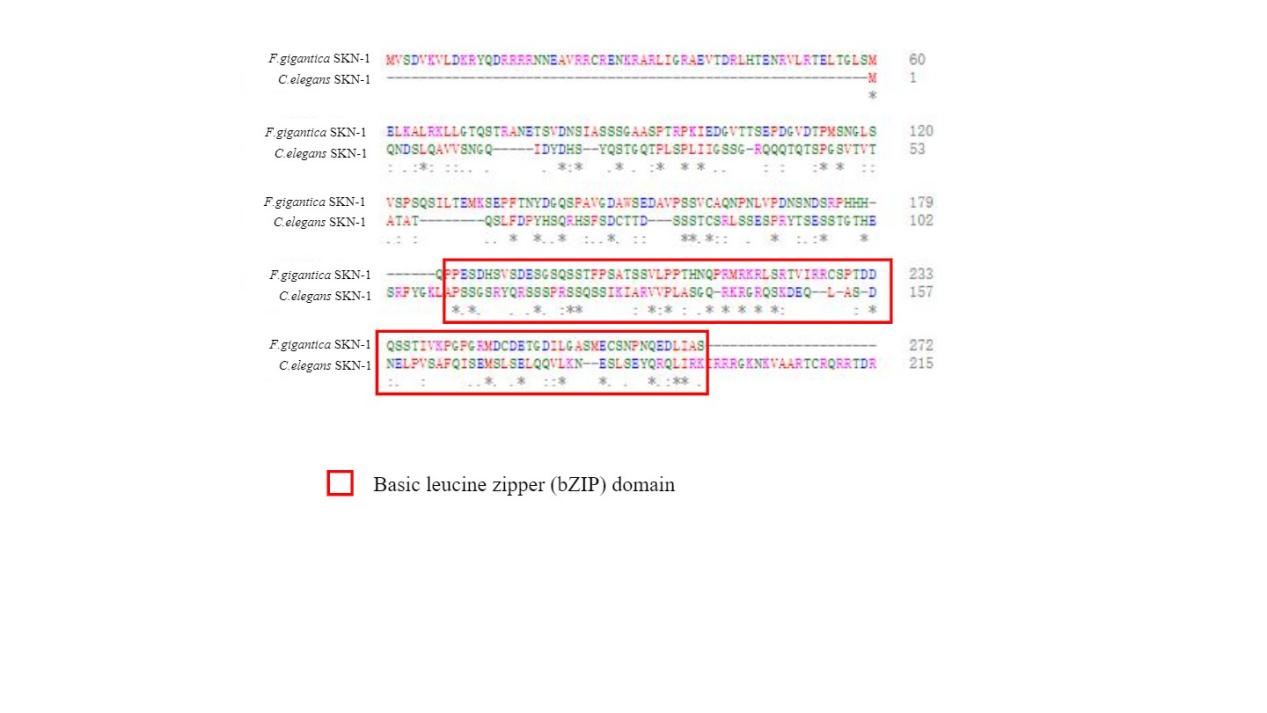
*

*Fg*HSF-1


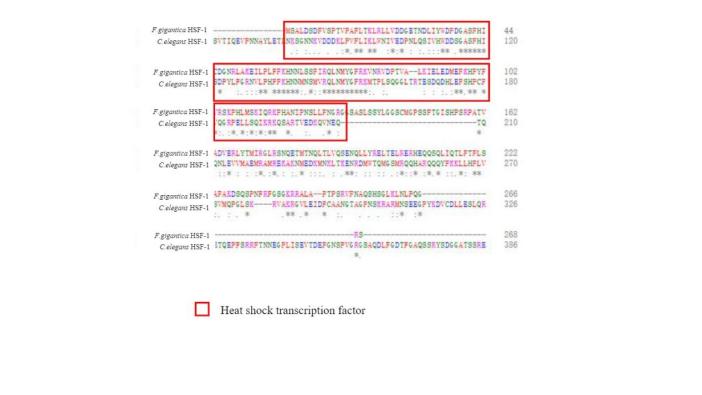


Fig. S3. A phylogenetic tree of insulin signalling pathway receptors in *Fasciola gigantica* and homologues constructed by the maximum-likelihood and maximum parsimony methods. **a.** Phylogenetic relationships of the IIS receptor in *F. gigantica* and homologues of 11 other species determined by the maximum-likelihood method. **b.** Phylogenetic relationships of the IIS receptor in *F. gigantica* and homologues of 11 other species determined by the maximum parsimony method. The bootstrap values are shown above or below the branches.

a


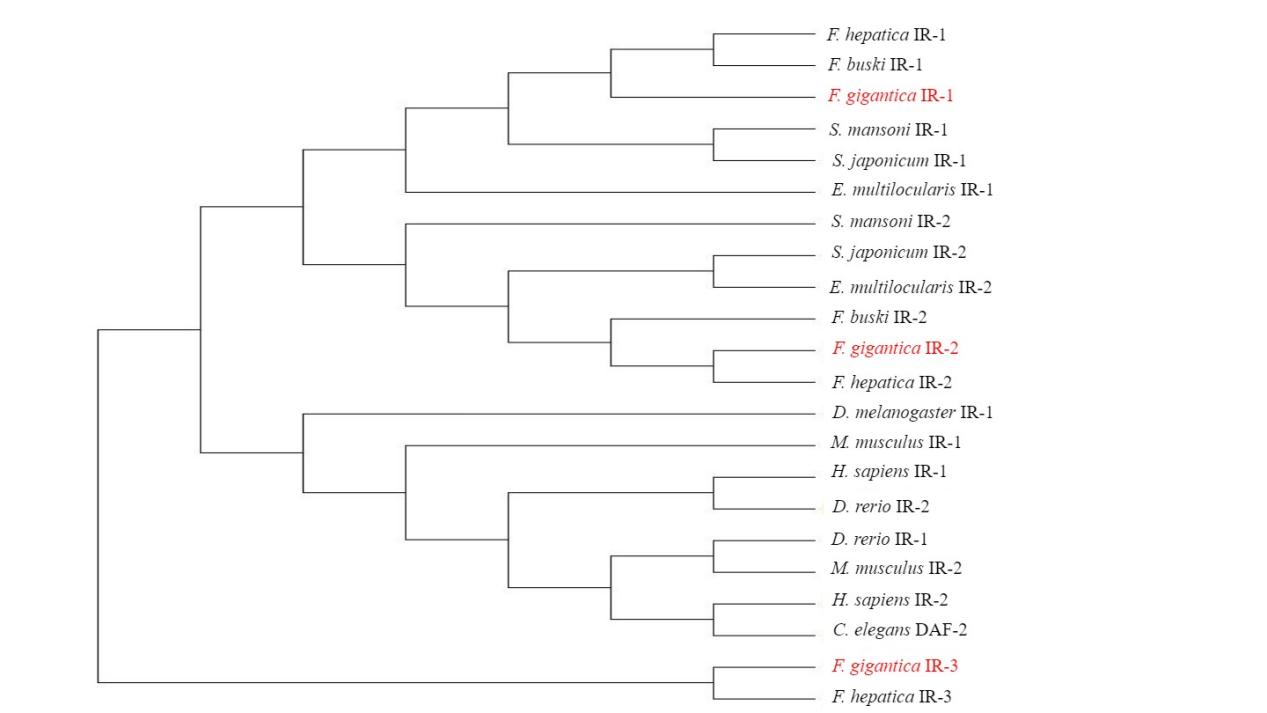


b


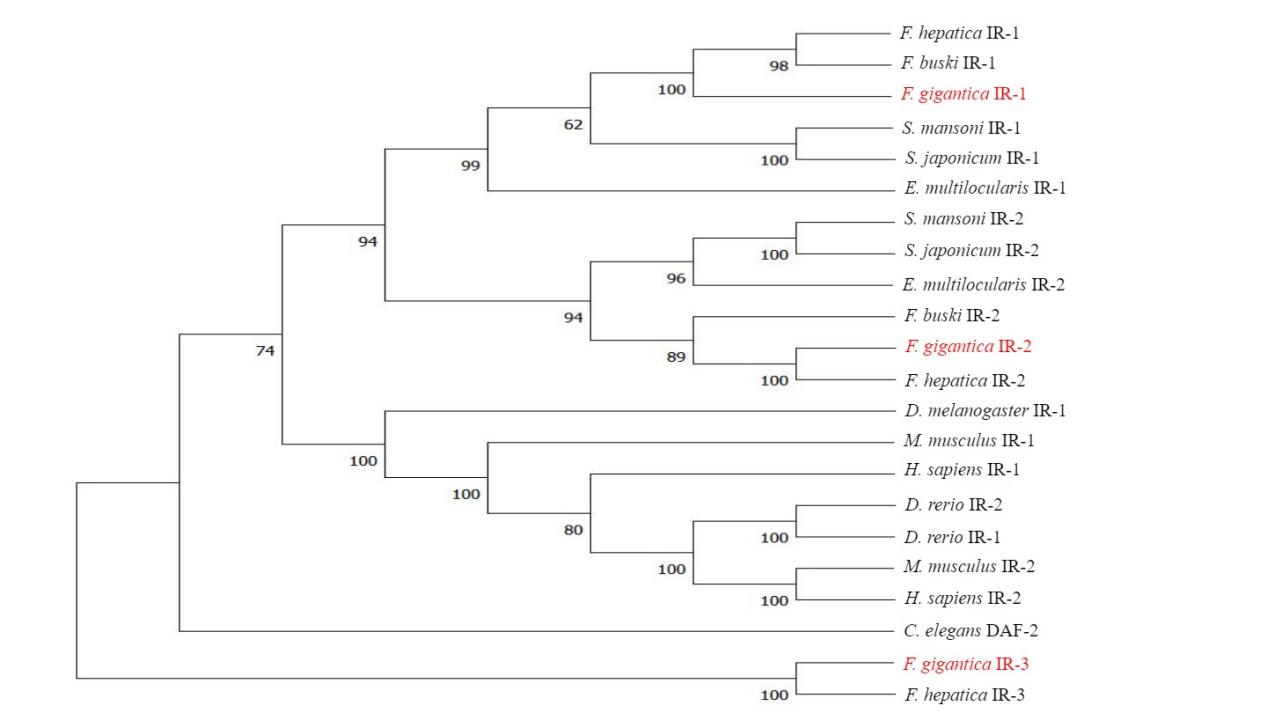

Supplement: Supplementary file 1 — Supplementary Material 1 [file 12917_2024_4107_MOESM1_ESM.docx]
