# Supplementary material for: An insulin-like signalling pathway model for Fasciola gigantica
Source: BMC Vet Res. 2024 Jun 8;20:252. doi: 10.1186/s12917-024-04107-7 (PMC11162077; doi:10.1186/s12917-024-04107-7)
Supplement: Supplementary file 2 — Supplementary Material 2 [file 12917_2024_4107_MOESM2_ESM.docx]

| Table S2. Primer sequences for genes amplification | | |
| --- | --- | --- |
| Target gene | Forward primer | Reverse primer |
| *Fgirs-1* | TTGACGTATCCACCGTGA | TCACTTACATGCACTACGT |
| *Fgilbp* | TTGTCTTTGTGGTACAGC | TATACTCGCTGGTGCGGT |
| *Fgakt-1* | CGGAACTCTGAATGTCC | GCCATTTAGGGGCCACTT |
| *Fgakt-2* | ACTCAACTGCGTTACTCG | CTAGGTTGCCAGCGGTAT |
| *Fgpdk-1* | TCAGTTGACAGCACCTCC | TGTCCGGTGAAGATCGGAC |
| *Fgsgk-1* | GGTCAGGCAAACTCTTC | GCCTTCGTTGAATGGTTCT |
| *Fgpp2a* | CCCATTTGCTGTTGTTGC | CTACGATTGAGCGTTGAGT |
| *Fg14-3-3ξ* | AGTCCTGGATCACCAATG | TCACTTGTCACCAGCAT |
|  |  |  |
| *Fgddl-1* | TTAACGAGGGTCGGGA | TGGCACTAGATCCTCAAT |
| *FgFoxo* | CCTCGTATATTCATCGC | CGCTACAGTTGGTTGTAT |
