# Supplementary material for: An insulin-like signalling pathway model for Fasciola gigantica
Source: BMC Vet Res. 2024 Jun 8;20:252. doi: 10.1186/s12917-024-04107-7 (PMC11162077; doi:10.1186/s12917-024-04107-7)

Fig. S1. Genes amplification. M: Marker, 1-10: coding genes of IIS pathway in *F. gigantica* (*Fgirs-1*, *Fgakt-1, Fgsgk-1*, *Fgilbp*, *Fgpp2a-1*, *Fgakt-2*, *Fgddl-1, Fgf*oxo, *Fg*14-3-3ξ, *Fgpdk-1*).


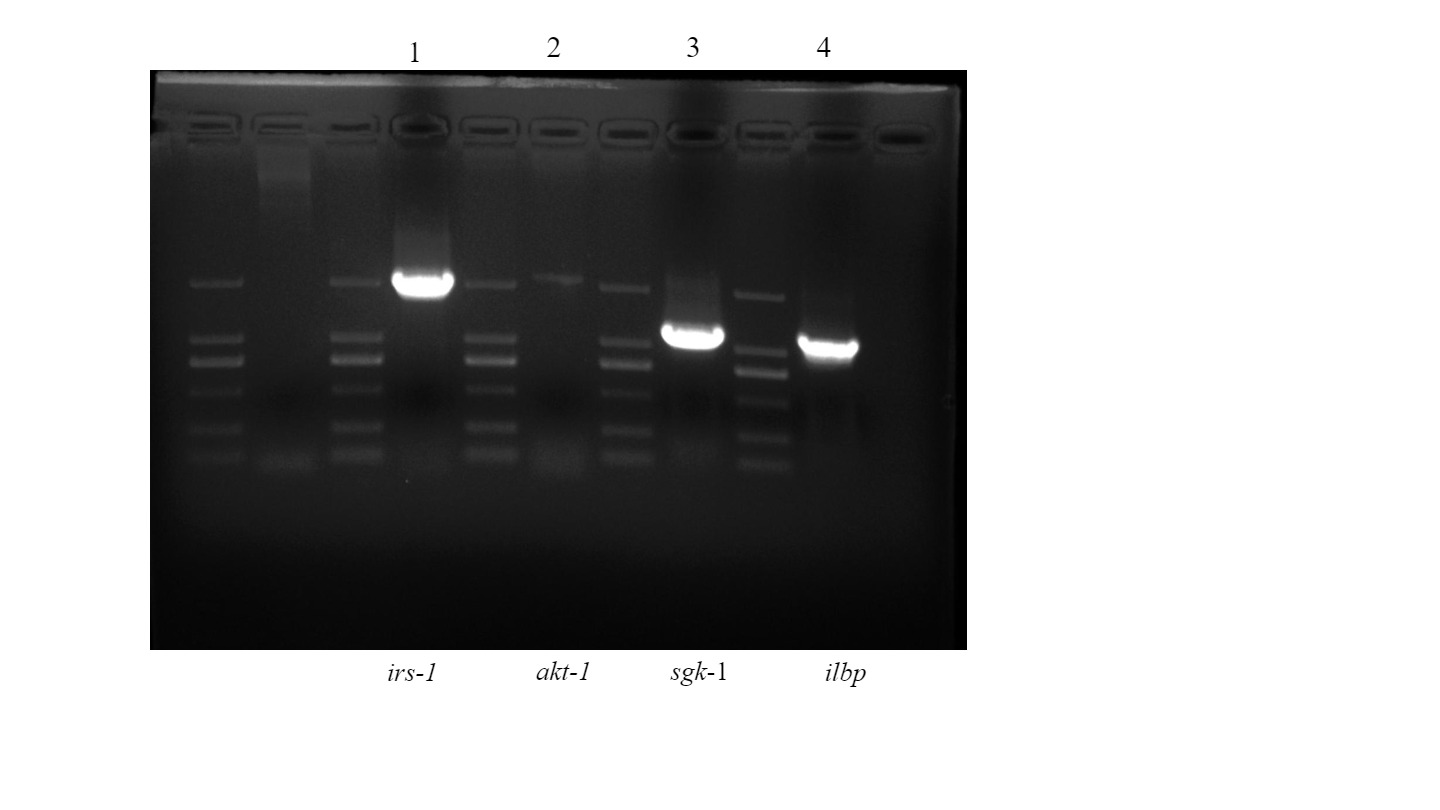

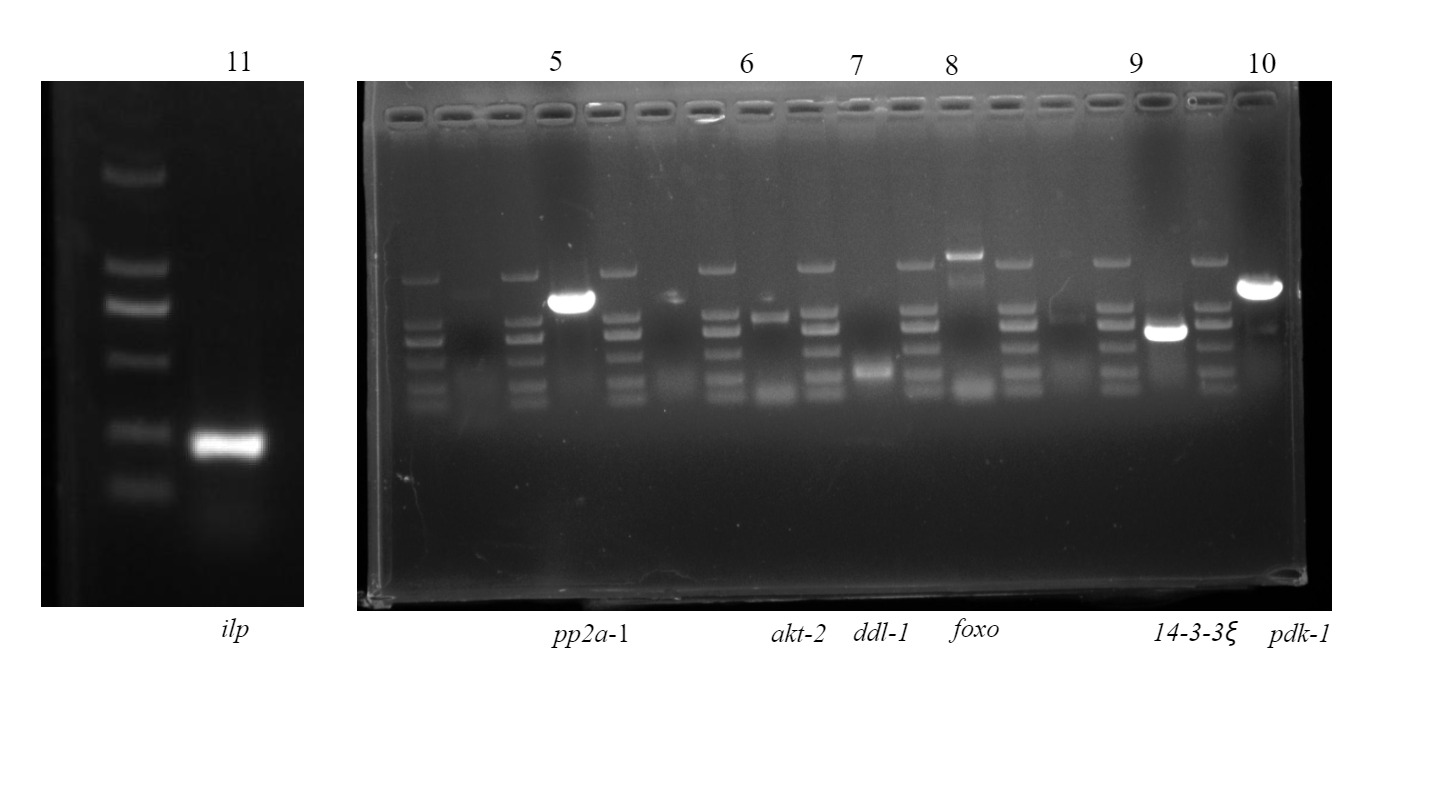

Supplement: Supplementary file 3 — Supplementary Material 3 [file 12917_2024_4107_MOESM3_ESM.docx]
